# Cumulative Effects of Adding a Small Group Intervention to Social Network Testing on HIV Testing Rates Among Crack Users in San Salvador, El Salvador

**DOI:** 10.1007/s10461-021-03160-9

**Published:** 2021-01-30

**Authors:** Julia Dickson-Gomez, Sergey Tarima, Laura Glasman, Wendy Cuellar, Lorena Rivas de Mendoza, Gloria Bodnar

**Affiliations:** 1 Department of Epidemiology, Institute for Health and Equity, Medical College of Wisconsin, 8701 Watertown Plank Road, Milwaukee, WI 53226 USA; 2 Center for AIDS Intervention Research, Medical College of Wisconsin, Milwaukee, WI USA; 3 Fundación Antidrogas de El Salvador, San Salvador, El Salvador; 4 Universidad Centroamericana José Simeón Cañas, San Salvador, El Salvador

**Keywords:** HIV, Crack, El Salvador, HIV testing, Social networks, Combination prevention intervention

## Abstract

The present study evaluates a combination prevention intervention for crack users in San Salvador, El Salvador that included social network HIV testing, community events and small group interventions. We examined the cumulative effects of the social network HIV testing and small group interventions on rates of HIV testing, beyond the increase that we saw with the introduction of the social network HIV testing intervention alone. HIV test data was converted into the number of daily tests and analyzed the immediate and overtime impact of small group interventions during and in the twelve weeks after the small group intervention. The addition of the small group interventions to the baseline of monthly HIV tests resulted in increased rates of testing lasting 7 days after the small group interventions suggesting a reinforcing effect of small group interventions on testing rates.

## Introduction

Researchers increasingly call for a combination of biomedical, behavioral, social and structural strategies, called combination prevention interventions, as the best way to reduce HIV incidence [1–4]. Combination prevention approaches are thought to be better at preventing HIV from sexual- and drug-risk behaviors because of the complexity of the causes of risk behaviors [5, 6], the difficulty of maintaining strict adherence to prevention behaviors [7], and in covering or reaching all key populations at risk for HIV with prevention materials [8]. For example, sexual risk among stimulant users is associated with several factors including sex for drug or money exchanges among men and women, the use of drugs to help cope with commercial sex work, sexual violence against crack users who engage in sex exchanges, poverty, and the stigma of drug use and sex work [9, 10].

Multiple biomedical prevention strategies (condoms, PrEP, circumcision and ART adherence) have been found to be superior in modeling studies compared to single strategies among sero-discordant couples in reducing the risk of HIV transmission over a number of years with ART adherence being the most effective single strategy, and PrEP and condoms the least effective when used alone, partially due to the difficulties of adherence [7]. Similarly, research with injection drug users suggests that a combination of approaches is needed to reduce HIV infection among PWID including: syringe exchange programs (SEPs); frequent HIV testing and linkage to HIV care; antiretroviral therapy initiation after infection; medication assisted therapy; psychosocial support; and pre- and post-exposure prophylaxis [11–13]. This is due to difficulties in coverage as well as barriers to strict adherence. Modeling projections suggest that very high coverage of ART, SEPs, and medication assisted therapy (MAT) in combination are necessary to reduce HIV incidence of PWID by more than 50%; very high coverage of single interventions is necessary to achieve similar effects, at a rate that has so far been unachievable. Otherwise, single interventions are unlikely to be effective (11). Researchers also believe that interventions must tackle social and structural factors that increase HIV risk, for example punitive laws criminalizing drug use and prohibiting safer injection practices, or include empowerment strategies to reduce stigma and violence against drug users, sex workers, and gender and sexual minorities to change the social context that contributes to HIV risk [6, 14–16].

While modeling studies suggest that combination prevention interventions are more effective than single approaches [4, 7], and some research has shown that combination prevention interventions that work at multiple levels (e.g. structural, social network and individual levels) are effective at reducing HIV risk [6], few studies have empirically looked at the cumulative effects of exposure to different components of combination HIV prevention interventions. One exception showed that a social protection intervention including child-focused grants, free schooling, teacher support, and parental monitoring were independently associated with reduced HIV risk among adolescents in South Africa [6]. However, social protection interventions in combination were shown to have a cumulative effect on reductions in HIV risk behaviors; for example, past year incidence of economically-driven sex dropped from 11 to 2% among girls who received a child grant, free school, and good parental monitoring [17].

The present study examines the cumulative effectiveness of a multi-level, combination HIV prevention intervention on HIV testing rates among crack users in San Salvador, El Salvador. Crack users in El Salvador are at high risk for contracting HIV. Our previous studies have shown an HIV prevalence of 4.9–7% (95% CI 2.3–9.8%), as well as low HIV testing uptake and high rates of risky sexual practices [18]. Seventy-two percent of our study participants reported sex with multiple partners in the past 30 days, 40% condomless sex with casual partners, 51% sex under the influence of drugs, and 33% sex in exchange for crack or money. Less than half reported having ever taken an HIV test prior to the study. As a result of these findings, we used a community-based participatory approach to develop Encuentro, a combination prevention intervention to address low HIV testing rates, high HIV and drug-related stigma, and sexual risk behaviors among Salvadoran crack users. Encuentro included community HIV testing, social network HIV testing, small group sexual risk reduction interventions, and community events, which were rolled out sequentially, in order to determine the individual and cumulative effects of different intervention components using an interrupted time series design. We have published results showing the effectiveness of the social network HIV testing component using dual incentives and peer referral, in increasing the number of monthly HIV tests, particularly among crack users [19]. An additional publication showed that exposure to multiple Encuentro components significantly reduced times participants had condomless sex [20]. The present study reports the cumulative effects of the social network HIV testing and small group interventions on the community’s rates of HIV testing, beyond the increase that we saw with the introduction of the social network HIV testing intervention alone [19]. We expected to observe increases in community HIV testing rates after the completion of each group cycle, as participants may seek out HIV tests themselves and encourage others to do so after considering their own risk behaviors and encouraging their peers to do so. In other words, the present study tests whether the small group intervention, which focused on reducing drug and sexual risk behaviors, increased the HIV testing rate among crack users in the community who did not directly participate in the small group intervention.

## Methods

**Fig. 1:**
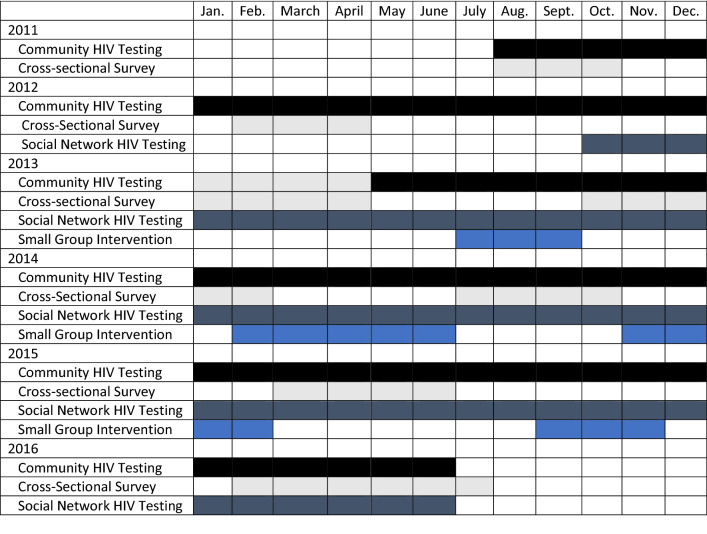
Encuentro intervention component and survey timeline

Project Encuentro consisted of four components designed to increase HIV testing and reduce sexual risk behaviors among crack users: rapid HIV testing in community sites; community events; social network HIV testing; and small peer-led group interventions for crack users. Intervention components were introduced sequentially and, once introduced, were continued until the end of the project. (See Fig. 1 for a timeline of intervention components and assessments.) The duration of Project Encuentro with the introduction of the four components was 42 months. The cumulative effects of intervention exposure on self-reported sexual risk behaviors was evaluated by seven cross-sectional surveys of crack users recruited through respondent-driven sampling. All seven cross-sectional surveys were identical and included the same measures. Results of these analyses were reported elsewhere [20]. The present study reports on the addition of the small group intervention (the last Encuentro component to be introduced) on monthly testing rates. Participants who either self-referred for an HIV test or came in with a referral coupon received from a peer took a small risk survey, and, if eligible, received coupons and instructions to recruit up to three peers for an HIV test. All participants who received any intervention components, i.e. an HIV test, participated in small group interventions, or participated in a survey, provided their written informed consent.

### Research Setting

All intervention and data collection activities took place in three low-income communities in San Salvador, including a community multi-purpose house in an informal slum settlement, a soup kitchen, and an AIDS Service Organization located in two different skid row areas of the city, adjacent to several informal settlements.

This study was conducted in collaboration with the Fundación Antidrogas de El Salvador (FUNDASALVA), a non-profit organization committed to the treatment of, prevention of, and research on substance abuse in El Salvador, which recruited and trained the field team. The team, which included six research associates and a coordinator, collected data, conducted the community HIV testing and social network testing intervention in the three communities, and helped facilitate small peer group HIV interventions with crack users along with a peer facilitator, i.e. a community member who formerly used crack.

### Community-Based HIV Testing

Rapid HIV testing was provided to anyone over the age of 18 who requested an HIV test regardless of whether or not they reported smoking crack. The availability of rapid HIV tests was advertised through posters placed throughout the communities and through word of mouth. Eight hundred twelve rapid tests were administered to people who self-referred during the project period.

### Community Events

Community events were held quarterly in each of the communities, beginning 6 months after the start of community-based HIV testing. Community events were planned in collaboration with community members and reflected themes of interest to them, for example, potential interactions of drug use with ART medication, violence against women, and drug use as a chronic brain condition, or in conjunction with other events, for example, setting up a table at a health fair organized by the community and the Ministry of Health. These events were open to all community members who were interested in attending, not just crack users, and were advertised through community leader invitation. Attendance at these events ranged from approximately 10 participants to 100 or more.

### Social Network HIV Testing

We introduced the social network HIV testing intervention after conducting the community-based testing for 18 months. Participants who initiated the referral chains, or “seeds,” were identified and recruited in collaboration with our community partners. Seeds, who were 18 years or older, had smoked crack in the previous month, and had not received an HIV test at the community testing sites in the previous three months, provided informed consent and completed a short questionnaire assessing demographics, whether they had smoked crack in the last 30 days, and substance use and sexual risk behaviors, and received HIV counseling and an HIV test. Risk factors included having had sex with a man (for men only), injected drugs, had multiple sexual partners, had been sexually assaulted, had sex with someone who had HIV, or had sex in exchange for sex or goods. While some of these behaviors do not carry a direct risk for becoming infected with HIV (for example, MSM are only at risk if they have condomless sex with a man of unknown or positive serostatus) we believed these criteria would enable recruitment of those who may be at high risk since MSM have one of the highest rates of HIV infection in Central America, with rates 33 times higher than the general population [21]. Only those who smoked crack and had one or more HIV risk behaviors outlined above were eligible to recruit others to take an HIV test.

To recruit peers to take an HIV test, participants were asked to list the initials of peers they thought were at risk for HIV and to describe these persons in terms of their sexual behaviors and substance use. This list of initials was not kept as part of any research or programmatic record and was used only to help participants to identify potential peers to recruit and to keep track of who the interviewer and participant were talking about when answering questions about peers’ risk behaviors. Lists were destroyed after participants recruited three peers or a month after their HIV tests in cases in which they did not recruit anyone. From this list, interviewers selected the network members who were described as using crack and engaging in sexual risk (as defined above) and gave participants up to three coupons to refer these members to take an HIV test. Participants were instructed that they did not need to use the real initials of participants. In addition, to maintain confidentiality, participants approached their peers to recruit them to the study and no information regarding what the participant had revealed was shared with potential recruits. Interviewers did not disclose eligibility criteria to avoid false reporting of eligibility. Counselors scheduled appointments for seeds to bring in their peers, asking them to inform their potential recruits of the confidentiality of the HIV test.

Referrals who were also crack users and reported past month sexual risk also obtained three coupons to refer members of the social networks who were crack users and engaged in sexual risk. All referrals who came with a referral coupon received $5 regardless of whether they were eligible based on crack use or sexual risk to refer their network members. Referrals who referred other peers for an HIV test received a $2 referral incentive for each referral, regardless of whether or not referrals were eligible to recruit members of their own social networks (i.e. referrals were not crack users who had engaged in HIV risk behaviors in the last 30 days). Finally, any person 18 years or older was still allowed to self-refer for an HIV test at the community testing sites. They completed the same short questionnaire and, if they reported crack use and HIV risk within the last 30 days, they were interviewed about their social network members and were eligible to receive the $2 incentive for each referral. Incentive amounts were not high enough to be coercive or influence drug use patterns as they were barely enough to cover the costs of one rock of crack in San Salvador. All participants who tested positive either in the community testing self-referral, component 1, or during the Social Network HIV testing phase, component 2, were referred to the Ministry of Health or a private lab for confirmatory testing and free HIV medical treatment. A total of 2815 tests were conducted after the Social Network HIV testing began, of whom, 2332 of the testers were crack users. Self-referred HIV testers who were not eligible to participate in the social network testing intervention were kept in a separate database for analysis from those who came with a coupon for HIV testing or were eligible as seeds to recruit others to the social network HIV testing intervention.

### Peer Network Small Group Intervention

After nine months of implementing the Social Network HIV prevention intervention, we introduced the small group intervention. To facilitate and recruit participants to the small group intervention, we selected a peer leader from each of the three community sites. Peer leaders were identified by community residents, were former crack users, and had extensive contacts with and a great degree of trust among crack users in the community. FUNDASALVA trained Peer Leaders in 10 3-h sessions held over two weeks to cover intervention activities, psychosocial theory, methods for ensuring participatory (as opposed to didactic) interventions, facts about HIV/AIDS, and methods for recruiting participants.

Peer leaders recruited active crack users who were invited to bring members of their drug using networks (i.e. people who know each other and use crack together) for each intervention cycle. Two peer network intervention cycles with 5–6 participants were held during intervention months, for a total of 30 cycles per study site. Participant cycles were timed not to occur during cross-sectional assessment periods to conduct both activities at once in the community sites. In other words, approximately six to eight small group cycles were completed before another cross-sectional survey was conducted. Five hundred and three (90%) out of 561 potential participants approached agreed to participate. Reasons for refusal included lack of time or interest. Each cycle consisted of three 2-h sessions held on consecutive days in the late morning. Peer leaders spent 10 h per week in recruiting and facilitating the intervention and were paid monthly stipends of $75 to compensate them for their time. Participants in the small group intervention received $5 and lunch for participation.

The small group intervention was based on the Transtheoretical Model [22] and was designed to move participants from pre-contemplation to action and maintenance of safer sexual behaviors (preexposure prophylaxis for HIV was not available in San Salvador so it was not included in the intervention). Sessions were interactive with ample opportunity to practice skills through role playing. Topics covered included information about HIV risk and protective behaviors, sexually transmitted diseases, condom use skills, condom negotiation, locations to obtain free condoms, identifying and avoiding situations in which risk behaviors occur, sexual rights and communicating with peers about harm reduction norms and practices. Ways of effectively communicating with peers about safer sex and HIV testing were practiced through role plays and participants committed to reducing their HIV risk and helping others through encouraging them to take an HIV test and use condoms.

A FUNDASALVA staff member was present and observed all peer network group intervention sessions to offer support to peer facilitators and ensure fidelity to the intervention. Staff presence was requested by peer facilitators to ensure safety and support facilitators when needed. Fidelity checks were completed for 15% of all sessions. Seventy-one percent of the sessions achieved 100% fidelity with mean fidelity at 94%. Attendance at small group interventions for all 3 days was also recorded and was used as an indicator of dose of the small group intervention in the analyses for this paper.

### Outcome Analysis: Impact of Small Group Interventions on HIV Testing Rates by Crack Users

To investigate the effect of introducing small group interventions on crack users’ HIV testing rates, we looked at the numbers of HIV tests conducted at the study sites, beginning with the introduction of the social network HIV testing intervention on October 1, 2012 through May 30, 2016. The last peer network small group intervention was on November 17–20, 2015. We converted HIV test data into the number of daily tests (for a total of 1336 days) and analyzed the immediate and overtime impact of small group interventions (SGI) during the SGI (3 days) and in the two weeks after the SGI. We used Poisson regression models, where the SGI dose was defined by the numbers of people who participated in each complete small group intervention cycle (3-day sessions), to determine whether the peer network small group intervention affected rates of daily HIV testing in the community and whether (and how long) this effect continued after each small group intervention was completed. The Poisson model was adjusted for the effect of location, year, and month. The impact of intervention is assumed to be mainly driven by the intervention dose with reduced impact over time. To evaluate the overtime pattern of intervention impact, the intervention dose was assumed to have differential impact during the SGI and two weeks after. Hence, three continuous predictors of intervention dose were investigated within the Poisson Regression model: “week1” (3 days during SGI), “week 2”, and “week 3.” Since SGI were separated by 2–3 weeks, longer impact of intervention was not estimable due to strong confounding between sequential SGIs.

## Results

**Fig. 2:**
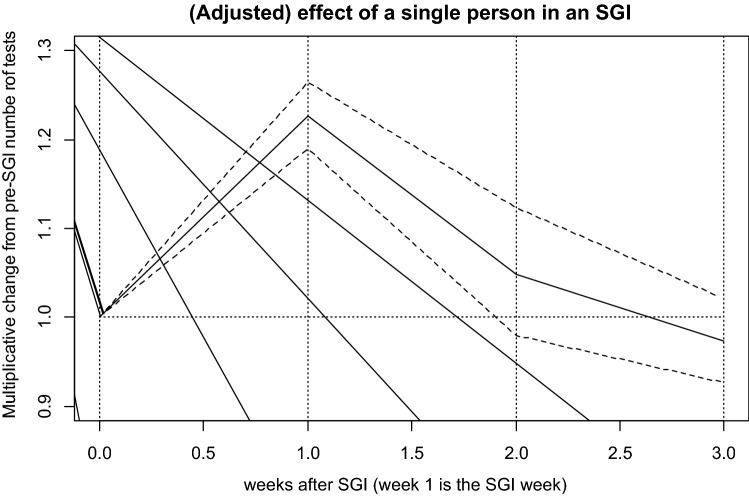
(Adjusted) effect of a single person in an SGI

In the adjusted Poisson regression model shown in Fig. 2 (adjustment for test location, year and month), each additional person in the small group intervention on a particular day during an SGI was associated with a 22.7% increase in the number of monthly tests by crack users (p < 0.0001). Similarly, each additional person in the small group intervention was associated with a 4.8% increase in daily number of tests by crack users in the first week following the intervention (p = 0.1755). Then, during the second week after an SGI there was a 2.7% decrease in the number of tests (p = 0.2725). The control variables, test location, year and month of the test were highly significant with omnibus p-values < 0.0001 each. The highest testing rate was observed right after the start of the intervention (October 2012), then significantly decreased. As shown in Table 1, Incidence Rate Ratios (IRR) are 0.2 (year 2013), 0.2 (2014), 0.1 (2015) and 0.1 (2016) when compared to monthly testing rates during October–December 2012 (the reference period). Testing rates were higher in Casa Esperanza location [IRR = 1.1] and lower at FUNDASIDA [IRR = 0.8] compared to the reference location “Casa Enmanuel”. There was some month to month variability with lower testing rates in December [IRR = 0.5] and August [IRR = 0.6] both months with weeklong national holidays, as compared to January (the reference month). The highest testing rates were observed in February [IRR = 2.2], July [IRR = 2.3], and October [IRR = 2.6] when compared to January.

**Table 1:** Incidence rate ratios of week, year, location and month on monthly testing rates

Predictor	IRR	95% CI		p-value
Week 1	**1.2267**	**1.1900**	**1.2646**	**0.0000**
Week 2	**1.0484**	**0.9791**	**1.1227**	**0.1755**
Week 3	**0.9731**	**0.9269**	**1.0217**	**0.2725**
Year = 2013	0.1991	0.1610	0.2463	0.0000
Year = 2014	0.1537	0.1226	0.1927	0.0000
Year = 2015	0.1079	0.0855	0.1360	0.0000
Year = 2016	0.0561	0.0400	0.0787	0.0000
Location = Casa Esperanza	1.0814	0.9424	1.2410	0.2649
Location = FUNDASIDA	0.8027	0.6914	0.9319	0.0039
February	2.2419	1.5531	3.2362	0.0000
March	1.5848	1.0746	2.3372	0.0202
April	2.0913	1.4408	3.0354	0.0001
May	1.8225	1.2481	2.6612	0.0019
June	1.7528	1.1918	2.5779	0.0044
July	2.3318	1.6225	3.3513	0.0000
August	0.5741	0.3488	0.9451	0.0291
September	1.8895	1.2944	2.7584	0.0010
October	2.5649	1.8035	3.6475	0.0000
November	1.1476	0.7897	1.6678	0.4704
December	0.5448	0.3590	0.8265	0.0043

Bold values are the IRR for the first, second and third weeks after the small group intervention, respectively

## Discussion

Results from this paper show the cumulative effects of a combination HIV prevention intervention on HIV testing rates among a community sample of crack users in El Salvador. Previous analyses of the intervention showed the effects of adding the HIV testing intervention on number of tests taken among crack users [19]. The social network HIV testing intervention resulted in an initial spike in the number of HIV testers, monthly HIV testing rates remained significantly higher and decayed more slowly than rates during the community testing period. The addition of the small group interventions to the baseline of monthly HIV tests resulted in increased rates of testing during the small group interventions. The greater the number of people in the small group interventions the larger the increase in testing rates, with one additional person per small group intervention causing an increase of 22.7% in testing rates during the intervention days. Thus, 5 people in an intervention group would increase testing rates among crack users by 58.7% during intervention days. These results came from models adjusted for testing location, month of the year and year of the intervention. These results suggest that small group interventions in combination with the social network HIV testing intervention served as a sort of booster to increase community testing rates among crack users. Presumably, this was a result of those who participated in the small group intervention going to take an HIV test and receiving coupons to recruit their peers to take an HIV test and so on. Small group interventions thus, in addition to decreasing the HIV risk and encouraging HIV testing among participants themselves, can have the community-wide effect of increasing HIV testing rates among high risk populations and should be considered as an effective tool in the seek, test and treat strategy.

Taking an HIV test may also result in testers reducing their sexual risk behaviors. The present study also used cross-sectional surveys of crack users recruited through respondent driven sampling to assess the effects of Encuentro components on HIV risk among crack users in the community. Participants did not have to participate in any intervention components as surveys were designed to discover the reach of the intervention into the community and community-level effects of the intervention on HIV risk. Participants who reported taking an HIV test in a community location or receiving a coupon and taking an HIV test (i.e. participating in the Social Network HIV testing intervention), as well as those who reported exposure to more than one Encuentro component, also reported reductions in condomless sex compared to those who received no intervention. However, the small group intervention did not reduce sexual risk behaviors on its own if participants did not also engage in HIV testing. Thus, small group interventions may work best by reinforcing the importance of HIV testing among a high-risk group of crack users which, in turn, may reduce sexual risk behaviors.

Project Encuentro is one of the first interventions shown to reduce sexual risk behaviors and increase HIV testing among crack users in a lower middle-income country (LMIC). Stimulant users, particularly in LMICs, have been neglected in HIV prevention interventions. In part, this is due to limited surveillance of stimulant users as an HIV risk group. Risk groups in surveillance are categorized according to the probable mode of transmission, e.g. injection drug use, men who have sex with men or participation in commercial sex work. Crack use carries no direct risk of HIV transmission, but as many studies have indicated, many crack users engage in sex work or direct sex for crack exchanges to support their crack habits [23–27], and many are victims of sexual assault [10, 28]. Our previous research showed an HIV prevalence rate of 5% among crack users, a rate similar to that found in the present study. Our study showed that it is feasible to conduct an HIV prevention intervention among crack users in LMICs and reduce their sexual risk. Our research also suggests that having multiple components can help in continuing to engage the community over time and sustain positive effects beyond those who directly receive the intervention.

### Limitations

The present study has limitations that should be considered in interpreting results. First, we used an interrupted time series analysis measuring the effects of the introduction of the last component on monthly testing results. This does not allow us to eliminate other potential causes for the increase in testing after small group interventions. A cluster randomized trial by community may partially resolve this problem. However, differences between communities and events that may have occurred within them during the study period would not completely eliminate the influence of other causes for the increase in HIV testing. However, in cross-sectional surveys we measured exposure to non-Encuentro HIV prevention interventions and HIV testing at locations other than the community sites found limited exposure to these.

An additional limitation was the necessity to stop small group interventions during cross-sectional surveys due to space and personnel limitations. Ideally, we would have continued small group intervention cycles without interruption once they were introduced. If we had been able to do this, we may have seen a stronger and more consistent effect on monthly HIV testing rates.
